# Developing textile sustainability education in the curriculum: pedagogical approaches to material innovation in fashion

**DOI:** 10.1080/17543266.2022.2131913

**Published:** 2022-10-31

**Authors:** Jane Wood, James Redfern, Joanna Verran

**Affiliations:** a Department of Materials, School of Natural Science, The University of Manchester, Manchester, UK; b Department of Life Sciences, Faculty of Science and Engineering, Manchester Metropolitan University, Manchester, UK; c Department of Natural Sciences, Faculty of Science and Engineering, Manchester Metropolitan University, Manchester, UK

**Keywords:** Textiles, fashion, sustainability, education, curriculum

## Abstract

The textile industry needs to adopt environmentally sustainable approaches to address ecologically damaging practices. Whilst driven by initiatives such as Textiles 2030, it is current students who will carry this agenda forward. This project investigated pedagogical approaches to develop sustainable textiles for the fashion design curriculum. Pilot studies, using bacterial cellulose (BC) as a material for millinery, revealed members of the public were prepared to experiment with this novel material, and BC was compatible with traditional hat-making techniques. A further study challenged secondary school students, based on an experiential learning model, to grow their own BC biofilm, exploring this as a sustainable apparel fabric. Initial attitudes of reluctance developed into acceptance once engaged in the practical activity. This study illustrates that with appropriate communication and education strategies, the principles of sustainability in fashion, and the acceptability of novel materials, can be engendered in different audiences.

## 1. Introduction

Initiatives such as the UN Sustainable Development Goals and Textiles 2030 are bringing the environmental impact of the practices of the apparel, fashion and textile industries into focus (UN, 2022; WRAP, 2021). Whilst many of these initiatives target legislation at a governmental or industrial law level, it is commonly acknowledged that real change in the industry will be implemented by the professionals of the future – current students (Abner & Baytar, 2019; Hiller Connell & and Kozar, 2012).

### 1.1. Sustainable fashion

The definition of ‘sustainable fashion’ is an elusive one, and open to interpretation (Henninger, Alevizou, & Oates, 2016; Mukendi, Davies, Glozer, & McDonagh, 2020). However, it is accepted that in fashion terms, an item that could be perceived as ‘sustainable’ may be described in terms such as ‘environmental, social, slow-fashion, reuse, recycling, cruelty-free or anti consumption / production practices’ (Mukendi et al., 2020).

### 1.2. Models for sustainability

Researchers have previously put forward two models for the conceptualisation of sustainable fashion, namely pragmatic and radical change (Burrell & Morgan, 1992). Pragmatic change involves the use of established methods (such as marketing and retail) to push the sustainable agenda, as exemplified by companies such as Patagonia (Patagonia, 2022). Radical change encourages action at a more fundamental level, addressing the accepted practices of the industry (Mukendi et al., 2020). Whilst both approaches are key in the implementation of sustainable practice in the fashion industry, this study focussed on the premise of radical change, challenging the concept of textile creation for apparel.

### 1.3. Textile materials

Most current fashion and apparel products are dependent on established textiles (e.g. synthetics such as polyester, nylon and elastane, or naturals such as wool, cotton and silk) and there is a fundamental need to understand the properties of the materials used to effectively design and develop products, which is already addressed to some degree in the secondary school and undergraduate higher education (HE) curriculum (Haq & Ite, 2022; Hiller Connell & and Kozar, 2012; Landgren & Pasricha, 2011). However, in recent years there have been several research studies focussing on developing textiles from alternative sources such as mycelium, food waste and bacteria (Wood, 2019) in an attempt to address the sustainability agenda. Most of these ‘fabrics’ are not yet in bulk production, nor are they freely available, thus fashion designers and product developers are not able to use such materials in their work. Nevertheless, the need to understand the properties of these materials is critical if they are to become part of the product developers’ and designers’ process in the creation of mainstream fashion.

### 1.4. Bacterial cellulose as an alternative textile

Bacterial cellulose (BC) is one such ‘alternative’ fabric, which has already been explored by some apparel developers (Fernandes, Gama, Dourado, & Souto, 2019; García & Prieto, 2019). It is developed as a biofilm on the surface of a growth medium (e.g. black tea and sugar) that has been inoculated with a bacterium. In the laboratory, BC is grown using lab-prepared media (which have been used previously in BC research), such as Yamanaka or Hestrin & Schramm (H&S) (Schramm, Gromet, & Hestrin, 1957; Yamanaka & Sugiyama, 2000). Other practitioners have adopted a more artisanal approach, using media such as pineapple juice, beer or black tea and sugar as growth support media to develop a BC biofilm for evaluation (Ha et al., 2008; Jarrell, Cal, & Bennett, 2000; Kumbhar, Rajwade, & Paknikar, 2015; Kurosumi, Sasaki, Yamashita, & Nakamura, 2009). *Komagataeibacter xylinus* is one of the most vigorous bacteria in its production of BC biofilm and is commonly used as an inoculant for the media mentioned above. It is found in Kombucha (used in the fermentation of tea drinks) (Chakravorty et al., 2016; Jarrell et al., 2000). Thus, the means for making and studying BC is freely accessible to the public for biofilm experimentation, enabling exploration of concepts of sustainability, usability and acceptability.

### 1.5. Kolb’s experiential learning model

It is widely accepted that there is a variety of learning styles and models (Reid, 2005). Coffield (2005) and Snider (1992) suggests that these are highly dependent on context and that neither students nor topics can be categorised; the models should only be used to develop frameworks and guidance and be adapted as each situation requires. The studies presented in this paper were developed in line with Kolb’s Experiential Learning Model (Kolb, 2022) which is based on learning through experience, reflection and further action. Whilst the model is more commonly applied to adult learners, in the context of this paper, the effect of this learning style is also discussed for secondary school students.

Therefore, the aim of this study was to evaluate the application of the experiential learning model with a variety of learners about the use of novel materials within the fashion industry.

## 2. Methods

Two events were conducted to gather data on the experiential learning model. The first event was a free-to-attend public engagement event, open to the public. The second event was a series of lunchtime clubs held at a local secondary school (children aged 11–12 years). The purpose of the two different events was to expose a variety of potential consumers to BC and evaluate the differences/similarities in their responses. The remainder of this paper provides the details of each event.

### 2.1. Public engagement event

A public engagement event, ‘Hats off to vLeather!’was held as part of Manchester Science Festival (www.scienceandindustrymuseum.org.uk/manchester-science-festival), at the Hat Works Museum of Hatting (www.stockport.gov.uk/topic/hat-works) in Stockport in 2017.

#### 2.1.1. Pre-session preparation

Tea culture medium was prepared by steeping ten tea bags (Yorkshire Black Tea, Bettys & Taylors Group) in 10 l of boiling water for 15 min. The bags were removed, and 1000 g sucrose added to the tea, stirring to dissolve. Once cooled the tea culture medium was placed into plastic containers (L 120 cm × W 60 cm × D 30 cm). The inoculum for the tea was taken from a purchased Kombucha pellicle (www.Happykombucha.com) which had been stored for 30 days at room temperature in a quantity of tea and sugar (as per the manufacturer’s instructions). 1000 ml of this liquid inoculum was added to the plastic container and the mixture stirred. The container was loosely covered with lightweight cotton sheeting fabric and stored at room temperature for 40 days to allow a BC biofilm to develop. After this time, a pellicle (i.e. biofilm fabric) had formed on the top of the tea culture medium, which was removed, rinsed with water and flat dried at room temperature for one week.

#### 2.1.2. Attendees

The attendees comprised 20 members of the public (ten per session, two sessions in total) who signed up for the event via the Manchester Science Festival website, on a voluntary basis. All health and safety requirements were implemented by the Hat Works Museum staff.

#### 2.1.3. Session outline

The two individual three-hour practical workshops were held on consecutive Saturday afternoons. Using pre-prepared sheets of BC (as described above), a milliner demonstrated some basic millinery techniques (blocking, steaming, and stitching) to the group. The group was then invited to look at some BC samples and choose the samples most appealing to them to create items of head wear using the demonstrated techniques. Observational notes were taken by the author on the participants activities, reactions to the BC fabric and the millinery pieces created by the participants. The observations were manually analysed to identify qualitative common themes raised by the activities, reactions, and verbal comments. Due to the small number of participants, no statistical evaluation was undertaken.

### 2.2. Lunchtime science club – Oldham Hulme Grammar School, Manchester

The school runs a science club on alternate Thursday lunchtimes, open to year seven (11- & 12-year-old) students. A ‘Grow You Own Fabric’ project was delivered across six weeks of the club.

#### 2.2.1. Pre-session preparation

Tea culture medium was prepared by steeping 5 tea bags (Yorkshire brand black tea) in 5 l of boiling water for 15 min. The bag was removed, and 500 g sucrose added to the culture medium, stirring to dissolve. In order to provide a comparison for the tea medium, a second culture medium was prepared in the laboratory by adding 2% glucose, 0.5% bactopeptone, 0.5% yeast extract to 5 l distilled water. This culture medium has been used previously in BC studies and is referred to as Hestrin & Schramm (H&S) medium. The H&S culture medium was prepared by adding 2% glucose, 0.5% bactopeptone, 0.5% yeast extract to 5 l distilled water.

Each of the tea and H&S culture media were placed in separate 1 l duran bottles (i.e five per culture medium) and autoclaved for 10 min at 115°C. Once removed from the autoclave, the media were left to cool to room temperature before use.

500 ml volume ‘kilner’ style preserving jars (www.kilnerjar.co.uk) were sterilised using sterilising fluid (www.milton-tm.com), prepared according to manufacturers instructions. The jars were submerged in the sterilising solution for 15 min and removed directly before use. Each jar was then filled with either 250 ml sterile tea or 250 ml sterile H&S culture medium (thus creating 20 jars of each from 5 l of prepared culture medium).

The inoculum for each of the tea or H&S culture media was taken from a Kombucha pellicle culture which had been stored for 30 days in a quantity of tea and sugar mixture (as advised by the pellicle manufacturer). A total of 40 × 25 ml sterile ‘universal’ tubes (25 ml total possible volume) were filled with 10 ml of the inoculum and stored at room temperature.

#### 2.2.2. Participants

The participants were 15 year seven students (a mix of genders) who regularly attended a lunchtime science club on a voluntary basis. The student group had been provided with basic details of the activities to ascertain their interest in the project and asked to commit to attending all 3 sessions across a 6-week period. Ethical procedures covering this event were put into place by the school; the author (JW) was always chaperoned by a DBS checked member of staff.

#### 2.2.3. Session outline

Each session was 20 min long (to fit in with lunch time scheduling).

Each session comprised a brief explanation of the practical activity and discussion with the students about the activity and the themes being addressed (Table 1). The students were asked to note their thoughts/reactions and the sheets were collected at the end of each session for thematic textual analysis. The students were asked not to put their names on the comments sheets to preserve anonymity. Any additional observations from the session were from the lead author. Analysis of the written text and author observations was qualitatively manually coded to identify themes. Where appropriate, the findings were visually presented to illustrate the relevant points.

**Table 1.** Outline of proposed themes in each session.

Session number	Theme	Question	Mode of answer
1	Initial thoughts on textile impact	Where do our clothes come from? What happens to our clothes when we have finished with them?	In class discussion/free text
	Expectations and learning outcomes	What do you think you will learn from this project?	In class discussion/free text
	Initial thoughts on bacteria – based fabrics	What do you think of making clothing/textiles from bacteria?	In class discussion/free text
2	Observations of biofilm development	What can you see happening in the jars? Can you measure the thickness of the biofilm? Can you describe the biofilm? What are your feelings about this?	Photography/free text
3	Observations of biofilm development	What can you see happening in the jars? Can you measure the thickness of the biofilm? Can you describe the biofilm? What are your feelings about this?	Photography/free text
	Perceived learning outcomes	What do you think you have learned during this project?	Free text

#### 2.2.4. Session 1

The school tutor and lead author gave a brief overview of the project and discussed the reasons why ‘alternative’ or ‘bio based’ textiles, created using microbiology techniques, were being explored.

The class were asked to consider their own clothing and their thoughts on where it came from, what it was made of and what they thought could happen to the clothing at the end of their use for it.

Each student was then provided with two pre-prepared kilner jars, one containing tea culture medium and one containing H&S culture medium, alongside two universal sample pots containing the inoculant mixture. Each student inoculated each kilner jar by pouring in the contents of one of the universal sample pots, closing (but not sealing) the kilner jar after inoculation.

Each jar was labelled with the student’s name and placed on a shelf in the biology classroom at room temperature (approx. 22°C).

The students were then asked to note in free text what they thought they would learn from this and the future classes.

#### 2.2.5. Session 2

Two weeks after the first session, each student was asked to examine their jars (opening the lids if required), record their observations of any changes since session one and take photographs with their phones or tablets. Students wrote down their comments and the sheets were collected at the end of the class.

#### 2.2.6. Session 3

Two weeks after the second session, the students attended another lunchtime session to discuss their observations of the changes in the jars, recording their comments using free text and photography.

This session coincided with the end of the school term, so the jars were collected by the author and kept off-site at room temperature (approx. 22°C).

After a further 2 weeks, the jars were photographed by the author and any resultant biofilms (pellicles) were removed. The biofilms were placed on a plastic sheet and allowed to dry on a desktop at room temperature for one week. The images and the resultant dried biofilms were returned to the school tutor to be given to the students on their return to school after 2 weeks. The teacher recorded comments from the students on the dried biofilms and these were verbally communicated to the lead author. The students were asked to reflect on the project and were invited to produce a poster documenting their learning. Three students participated in this exercise.

## 3. Results

### 3.1. Public engagement event

The author gave a brief verbal overview to the audience of the problems the textile industry was facing and therefore the need to look at alternative textiles that have less environmental impact. The groups agreed that there was a need to look at alternatives, but during the discussion, the author observed that all participants acknowledged they had little understanding of what this would mean in terms of changing manufacturing practice in the textile industry. The author described the impact of textile effluent on the natural environment and explained how BC sheets were grown, thus eliminating liquid pollution.

However, the use of bacteria in fabric production was not well received, with comments such as ‘*That’s disgusting*’ and ‘*the thought of it …  … it makes my skin itchy*’. Whilst some members of the group acknowledge that the BC offered a novel method to fabric production, none of the group expressed an interest in wearing clothing made from such methods.

**Figure 1.**
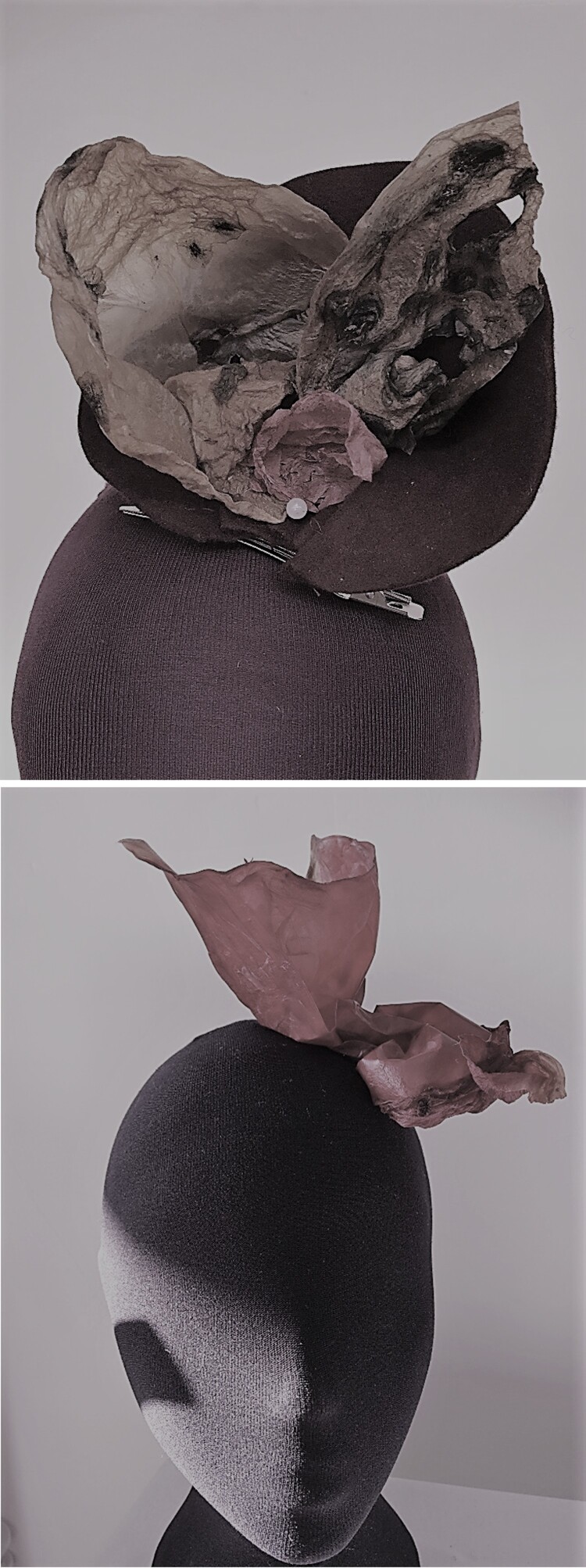
Examples of head wear created from bacterial cellulose.

The milliner then gave a practical demonstration of steaming and blocking using a traditional wool fabric and BC fabric. The participants were invited to take samples of BC to create items of millinery. The reactions to physically handling the fabric were ones of surprise ‘*it feels a bit like thin leather*’ and ‘*it’s more flexible I expected*’. None of the participants expressed any reservations about using the fabric and all were enthusiastic about expressing their creativity through the material. All participants created a headwear piece to take home with them (Figure 1). At the end of each event, whilst the participants still felt it was unusual to consider fabrics made from bacteria, all agreed that they had more open minds to consider alternatives to traditional textiles.

### 3.2. School lunchtime club

#### 3.2.1. Initial reactions

The first lunchtime club involved a brief discussion with the students about their initial comments and feelings regarding the issues of sustainability in the clothing and textiles industry.

#### 3.2.2. Q1: where do our clothes come from/what are they made of?

**Figure 2.**
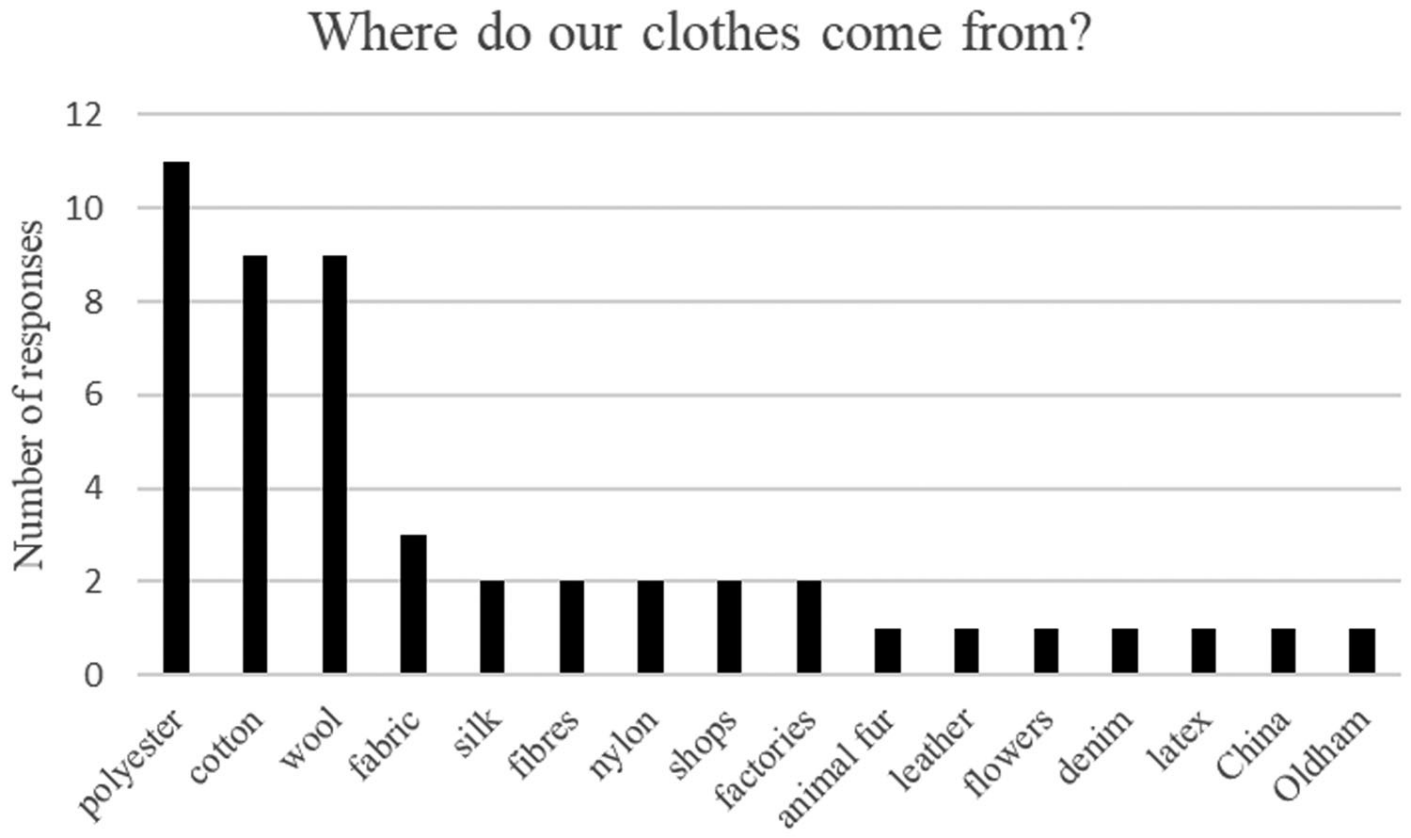
Students’ perceptions of fibre type / origins of clothing.

This question was first discussed generally in the class. Whilst some students did not feel confident to verbally answer the question, some gave very literal answers (‘Oldham’/‘China’) but when asked, they explained that they thought these were the main locations of factories manufacturing clothing. All students were asked to write their comments in answer to the question, with several giving more than one answer. Figure 2 shows an overview of the students’ written thoughts regarding the origin of their clothing (where relevant, multiple answers are included in the figure).

There did appear to be a broad knowledge of fibre types in the student group and the broad spectrum of fibres and sources mentioned can be seen in Figure 2. The most common answer was ‘polyester’. This is not surprising, polyester is the most common synthetic fibre used in apparel (Objective, 2022). However, this answer could also be attributed to the students looking at the labels in their clothing (they were all wearing polyester school uniform blazers). Wool and cotton featured highly in answer to this question; these are also some of the most commonly used fibres in apparel, jointly accounting for approximately 25% of the global clothing market (Objective, 2022).

#### 3.2.3. Q2: what happens to our clothes when we have finished with them?

**Figure 3.**
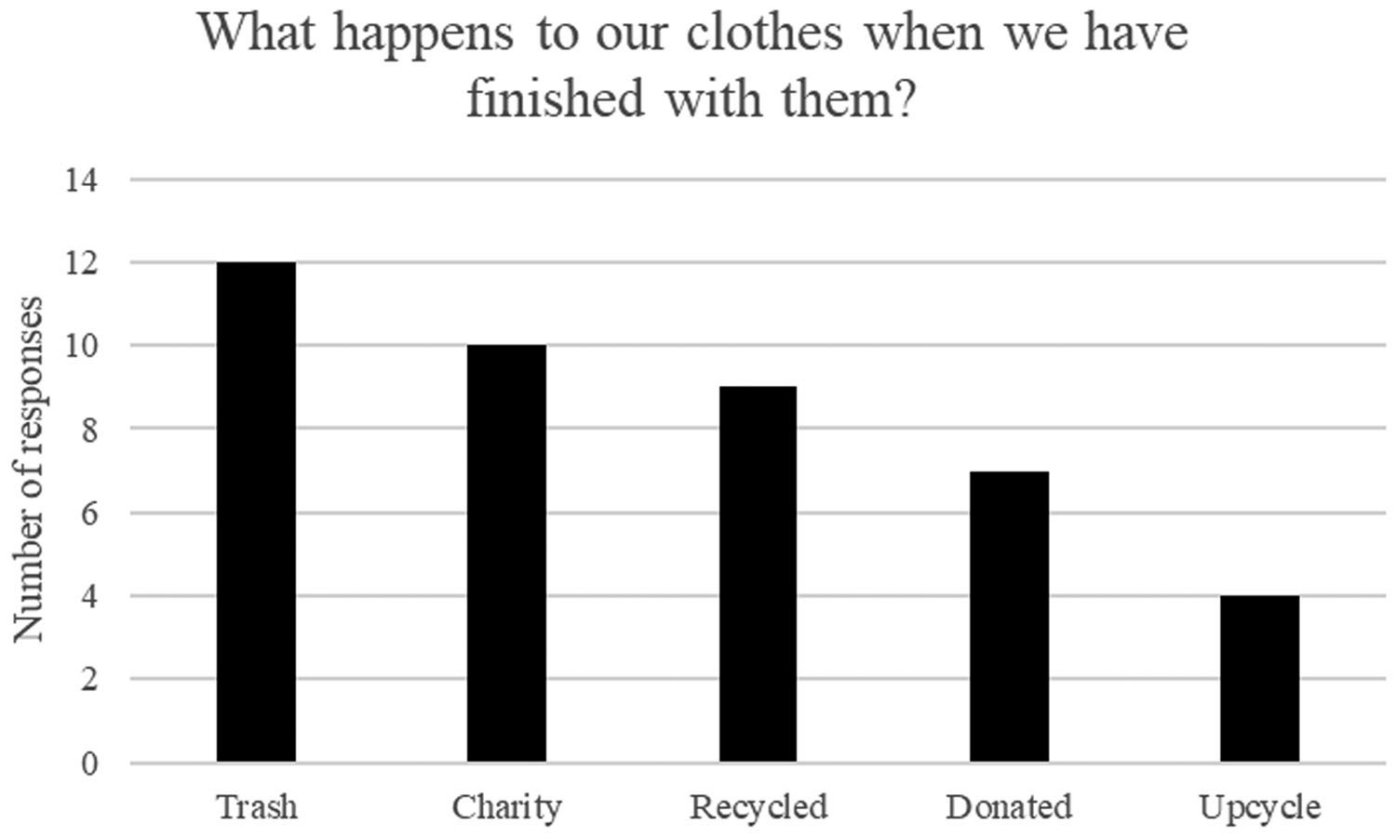
Students’ comments on ‘end of life’ clothing destinations.

This question elicited far fewer responses. Students generally agreed on 5 destinations for used clothing (Figure 3). Some students provided more than one answer to this question – all answers are documented in Figure 3. The majority felt that some of their clothing was thrown away (‘trash’) and not reused, but also acknowledged that clothing could be donated to either a family member or peer (donated) or passed on to a charity organisation (charity). Interestingly, only around a quarter of the students mentioned upcycling. When this was further discussed with the class, the students were clear in their thoughts that once the clothing had reached the end of its useful life in its current format, its next destination was trash. There was no understanding of recycling of the garment or the textile in this context. When asked about the response of ‘recycling’, the students could not define difference in this term from ‘charity’ and ‘donated’. It is therefore assumed that the responses of ‘charity’, ‘donated’ and ‘recycled’ have similar meanings in the student’s realm of understanding.

#### 3.2.4. Q3: what do you think you will learn from this project?

This question aimed to explore any preconceptions the students had to using ‘alternative’ textile sources. They were clear in their expectations with almost all answering in the theme of ‘how fabrics are made’ or ‘how to make eco-friendly fabric’ (only one student said they thought BC as an alternative fabric was unworkable). This was encouraging as it showed the students had an interest in learning more about new textile sources.

#### 3.2.5. Q4: what do you think of making clothing/textiles from bacteria?

Whilst students had expressed an interesting in find out more about alternative fabric production in Q3, Q4 illustrated their reservations regarding using bacteria to create clothing fabrics. Initial responses included concerns around the smell and texture of the fabric, with others showing repulsion regarding the use of microbes in textile generation. This opened a wider discussion with the school’s biology teacher who linked the presence of microbes and the term biofilms to topics the students had already covered in the year 7 curriculum (plaque on teeth, food spoilage). This led the students to reconsider their initial thoughts (‘*I think we will learn that bacteria can be useful sometimes apart from health*’) and whilst some still had reservations regarding the fabric samples they had seen (‘*don’t use bacteria, it’s uncomfortable, hard and stiff’* and ‘*it’s kind of gross’*), most of these opinions were countered with more positive comments (‘ … *.the use of bacteria is innovative and clever’; Making clothing out of bacteria is a brilliant idea; making clothes out of biofilm would be a very good for the environment*). The students could make a clear and positive link to the benefits of this type of textile in terms of its impact on the environment, with most students mentioning terms such as ‘*eco-friendly*’, ‘*pollution* (reduction)’ and ‘*environment*’ in their comments.

#### 3.2.6. First impressions of biofilm development

**Figure 4.**
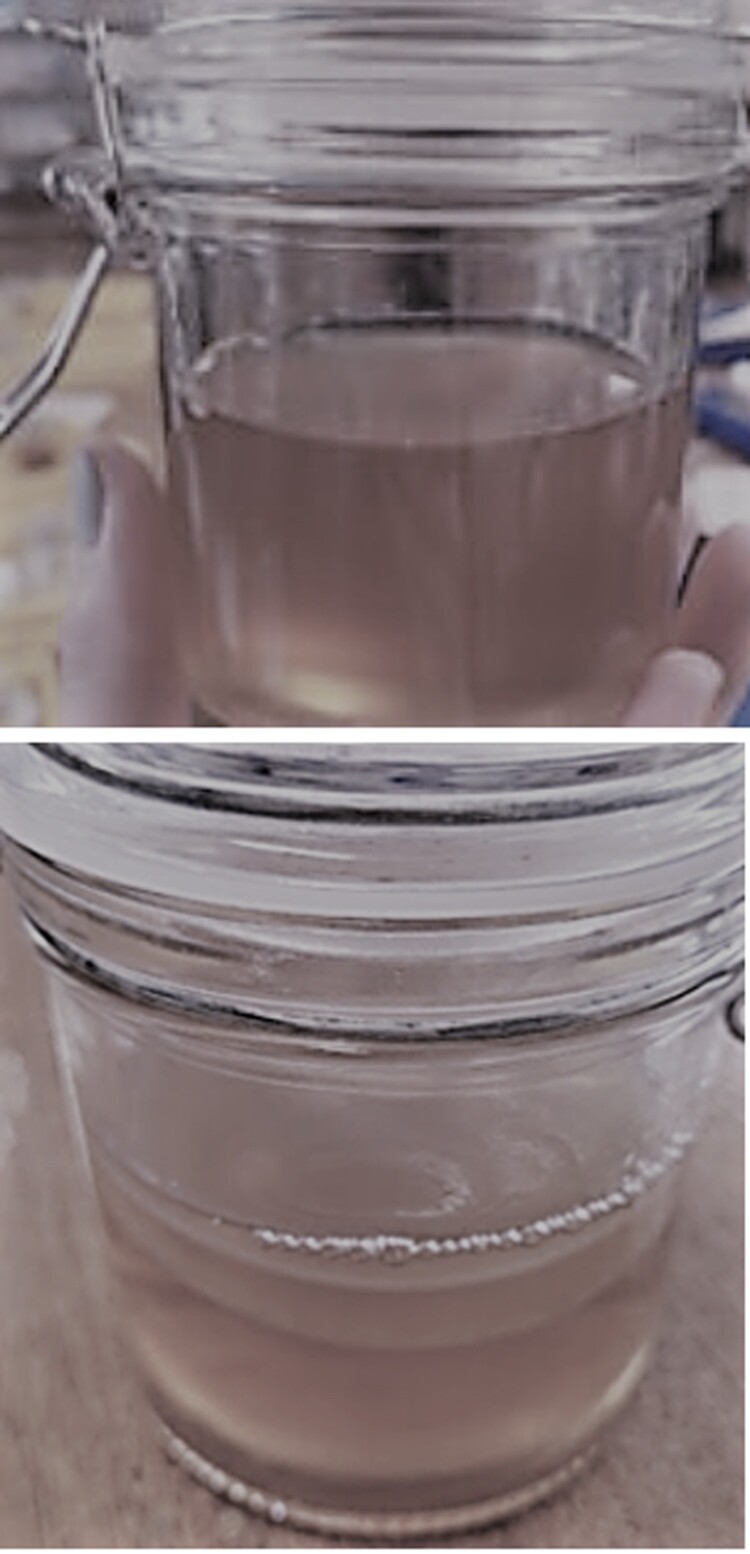
BC biofilm development in tea culture medium. Session 2: A thin, transparent biofilm has developed on the surface of the culture medium. Session 3: Thicker, more opaque biofilms developed with multiple layers noted in some jars.

The second session was designed for the students to monitor the progress of the biofilm development in the jars. In the jars containing the inoculated tea culture medium, all students could see the development of a transparent film on the surface of the culture medium, measuring between 1 and 5 mm in thickness (Figure 4). The students commented on the jelly-like consistency of the biofilm, and many mentioned the vinegar-like smell of the culture medium when the pots were opened.

**Figure 5.**
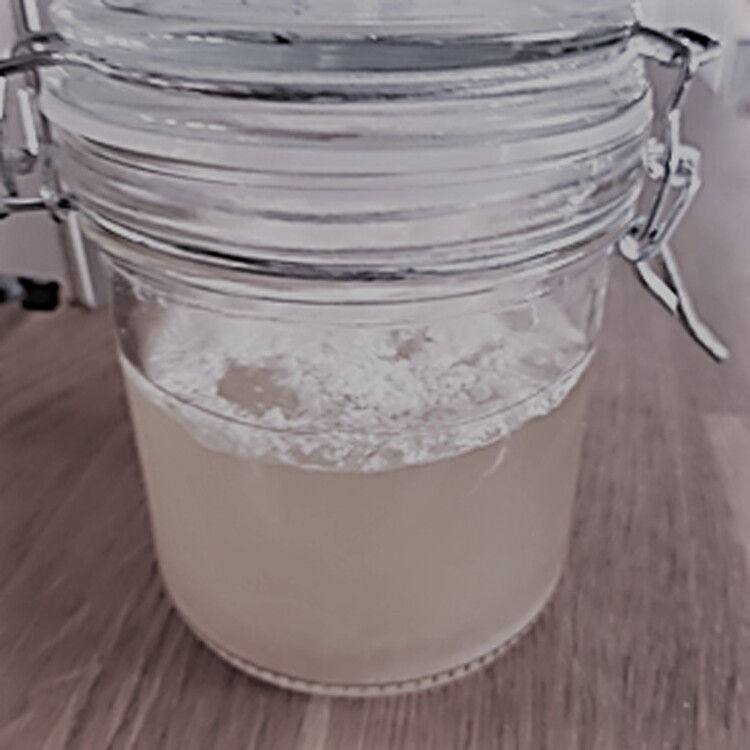
BC biofilm development in H&S culture medium.

Many of the jars containing the H&S culture medium had not developed a biofilm and there were instances of contamination with the development of obvious fungal growth in these jars (Figure 5). In these cases, the students were advised not to open the jars (to prevent the release of spores). This again engaged the students in conversation with their biology teacher regarding the development of fungal spores into mould, linking to previously covered year seven curriculum topics.

All the jars were replaced on the classroom shelf and stored in ambient conditions until the next session.

#### 3.2.7. Further biofilm growth

Session 3 was the last formal session with the students. In this session the students made observations on the development of the biofilms. Most of the H&S culture medium jars had developed fungal growth contamination, and these were disposed of appropriately (Figure 5). However, three of the H&S jars had developed biofilms and the students noticed vigorous development of these; the biofilms were opaque, white in colour and approximately 10 mm thick (Table 2).

**Table 2.** BC biofilm pellicles removed from jars (each pellicle 6–7 cm diameter).

	Wet	Dry
Tea culture medium	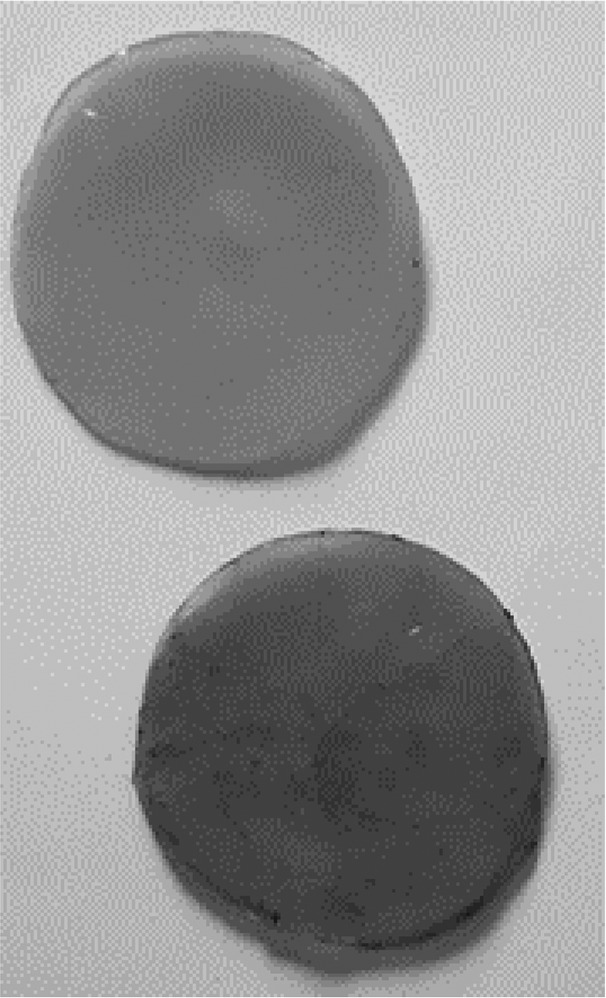	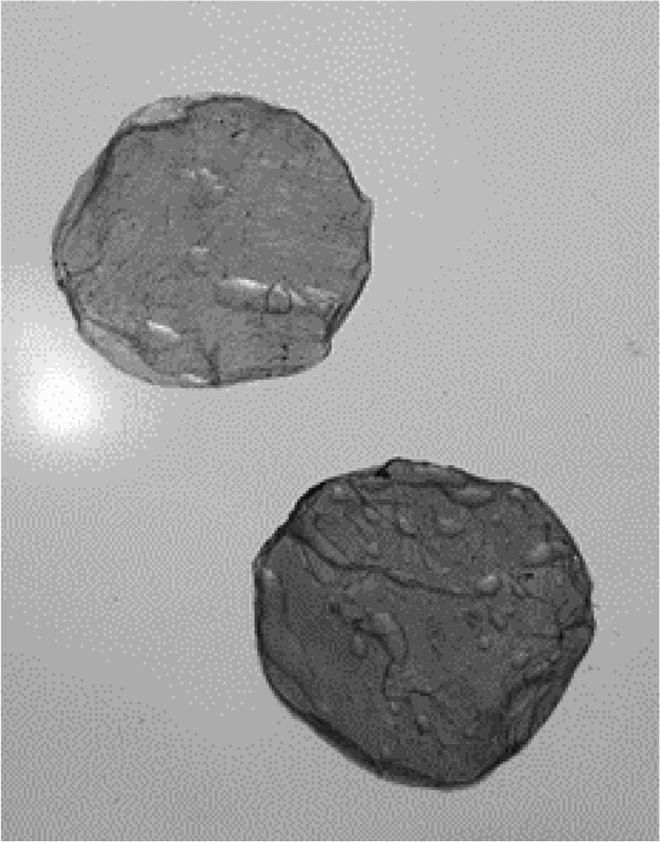
H&S culture medium	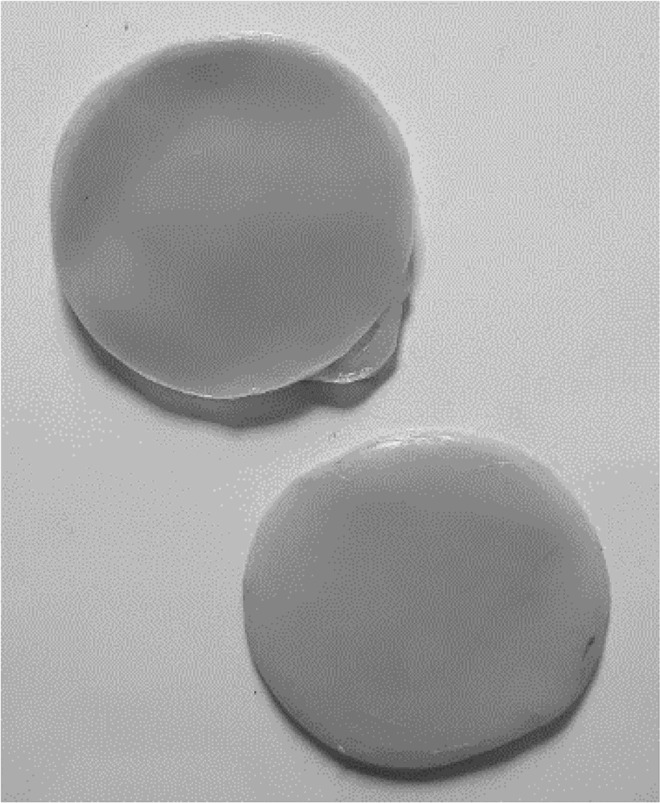	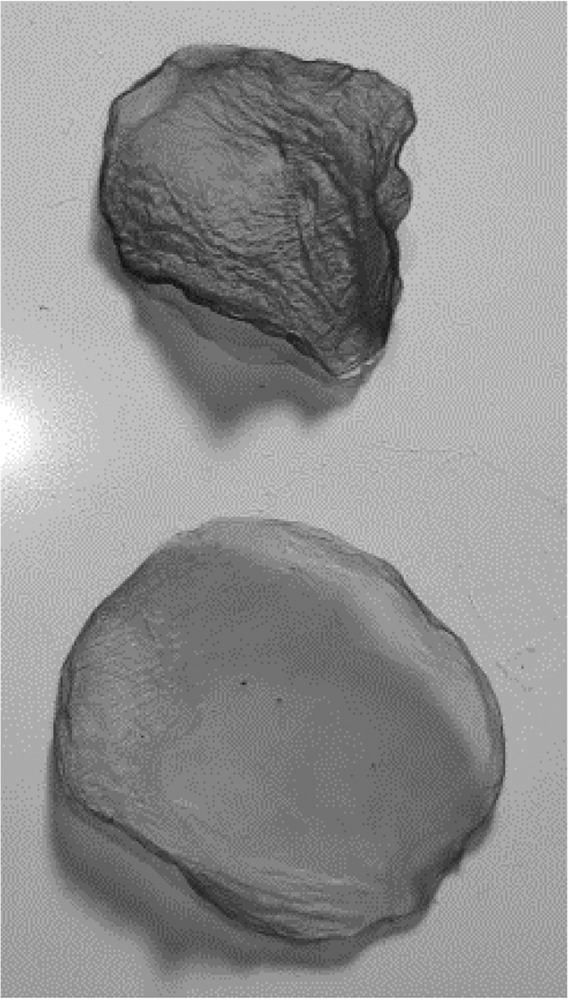

Most tea culture medium biofilms had developed further, with students noting bubbles in the liquid underneath the biofilm, and biofilms measuring approximately 10 mm in thickness (Figure 4). It was also noted that some jars contained multiple thinner biofilms, rather than one thicker sheet (Figure 4).

Before the biofilms were removed from the jars for drying, the students were asked to record in free text what they felt they had learned from the project. All the students agreed the fabric could be considered environmentally friendly (‘*the biofilm is eco-friendly and can be broken down’, ‘I think it is smart that we have come up with a way to make an eco-friendly material for clothes’*). They all commented that they had realised the need for changes in the textiles industry and several mentioned that they were now more aware of the polluting effect of textiles and clothing (*‘ … . pollution is becoming more in the world*’). However, there were several concerns raised about the useability of the biofilm they had created, once they received the dried biofilms (*‘its crumpled and smelly’, ‘it’s weird’*) with some students suggesting this could be a barrier to public adoption of this type of material (‘*I think this would cause a lot of controversy with members of the public because they would be disgusted …  …  … . If we could alter the textures of the fabric the bacteria will make people would be more convinced to buy them*’).

**Figure 6.**
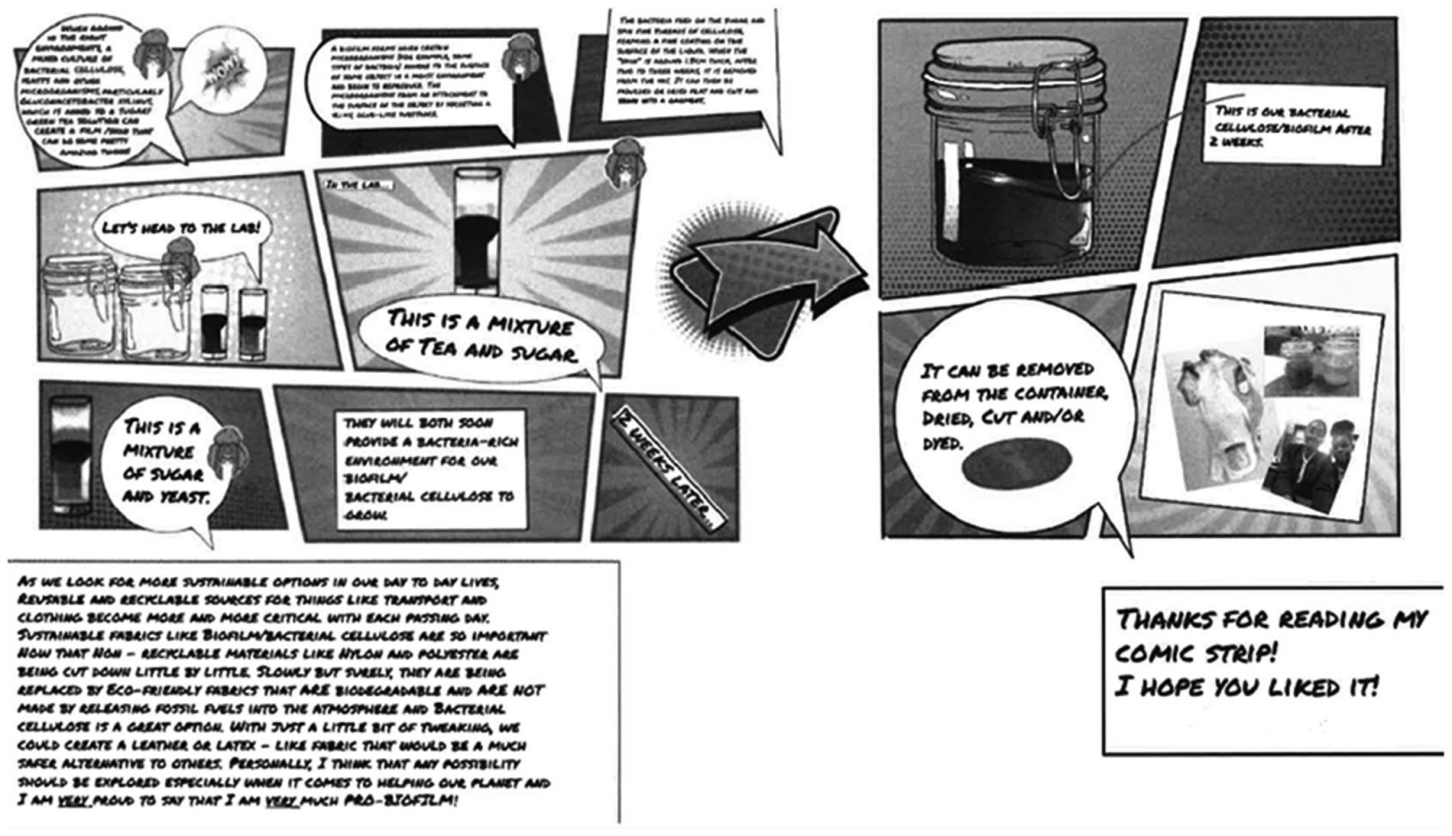
Poster produced by student detailing the project and learning journey.

The three posters submitted detailed the students understanding and illustrated their engagement with the biofilm development process. Figure 6 is an example of one of the posters; the student adopted a ‘comic strip’ approach to detail the process.

## 4. Discussion

Both the public engagement event and the school science club events showed that there was some awareness of the polluting nature of the textile industry and the impact that fabrics currently used in clothing could have on the environment. Nevertheless, the school students’ comments highlighted the need for wider education around recycling of clothing beyond its initial use; there was little appreciation of reusing the textile or componentry of garments for different end uses beyond clothing.

Furthermore, there was little awareness around alternative approaches; none of the participants (across all events) had heard of using bacteria or biofilms to create fabrics and there was reluctance across all the groups to consider this approach when put forward as an idea. This receptiveness to new ideas sits with Kolb’s learning cycle which suggests that the learner is more likely to engage with the ideas put forward if this can be grounded in concrete experience, rather than presented as a series of facts or potential solutions to a problem (Kolb, 2022). Discussion showed reluctance to accept BC as an alternative fabric, but the physical activities (experiential learning) gained the trust of the participant, opened pathways to enquiry and ultimately enabled more engagement in consideration of BC as a potential future textile. A further illustration of this is the behaviours observed in the public engagement event: the participants were very happy to use the BC ‘fabrics’ to create headwear, despite voicing reservations at the beginning of the session when the concept was first presented. In a similar way, the school students used terms such as ‘*eeewwww*’ and ‘*disgusting*’ when they first discussed BC as an alternative, and after the first 2 weeks of growing, with students commenting on the vinegar-like smell of the jars. The author explained that this was entirely normal in this type of biofilm development and had been noted many times in other research (Jarrell et al., 2000; Chakravorty et al., 2016; Dufresne, 2012; Mohite and Patil, 2014; Jayabalan et al., 2014). Once engaged with the physical act of growing their own fabric, they started to use words such as ‘*cool*’ and ‘*innovative*’ in their descriptions. There were some reservations voiced at the end of the process, once the dried biofilms were reviewed, but all students could see the benefit of further exploration of this type of material and even came up with some suggestions of what they thought was needed to encourage mainstream adoption (* …  …  … . If we could alter the textures of the fabric the bacteria will make people would be more convinced to buy them*’). Despite Kolb’s learning theory traditionally being applied to adult learners (Kolb & Kolb, 2022; Reid, 2005), our study illustrates it is also applicable to secondary school students.

Whilst this study illustrated the benefits of the experiential learning model for the use of bacterial cellulose, the activity itself presented some difficulties. The length of time taken to grow the biofilm was approximately five weeks; this could be prohibitive in some educational settings. Additionally, contamination of some of the jars was observed which could also cause issues with cross contamination and subsequent safe disposal. This could be mitigated by the pre-preparation of materials (as per the public engagement event), or by providing the materials and instructions for participants to grow the material at home (and how to dispose of any contaminated jars), documenting the growth cycle in a similar way to the school science club, and bringing the dried material into class for further exploration and discussion. This could also present the possibility of linking across subjects such as design and technical performance to enhance learning opportunities.

Brandenburg and Wilson (2013) suggest that some students hold the belief that learning is something that is done to them; it is a passive activity that is the responsibility of the teacher and not the student. In our study, the actions of all groups suggest that physical activities in which the participants were encouraged to explore the BC, both from a growing and an application viewpoint, elicited better engagement and promoted deeper discussion than the initial didactic presentation of the concept of biofilm fabrics. Brandenburg and Wilson (2013) also suggest that listening to the student voice and adapting to their needs is critical; the findings of this study support this idea; initial rejection of BC turned to acceptance once the approach to learning changed. This is a concept that Brown, Roediger, and McDaniel, and A (2014) support, stating that a mixed approach deepens learning and allows adaptation of knowledge to different scenarios, which was observed in our study.

## 5. Conclusions

This study observed the perceptions of samples of the public and year seven students on the use of alternative textiles (BC) in the fashion industry using a pedagogical model based on Kolb’s experiential learning theory. Whilst the participants across all groups were initially reluctant to consider the use of BC due to its mechanism of manufacture (grown from bacteria), the attendees of the millinery workshop were happy to use the material once they had handled the samples, whilst the year seven students engaged in the process of ‘growing your own fabric’ during the lunchtime science club, acknowledging the need for alternative textiles.

The study illustrated the difficulties of conveying the concepts of alternative bacteria-based fabrics using didactic methods; for both audiences, the material was rejected as a possibility when described in this way.

Lessons were learned for those planning future events – for example, the H&S medium appeared more susceptible to contamination in this study and could be omitted. In addition, the length of time required to grow the biofilm could exclude this activity from some events, although prefabricated samples could be provided, or, the participants could take the incubation mix home for growth and subsequently feedback observations to the research team (authors). This could also be built into the curriculum and timetabled as part of a structured syllabus.

There is a need to promote radical change in textiles and apparel to address the effects of the industry on climate change. Our study illustrates that alternative textiles such as BC can be used as engaging and illustrative examples by students and the public if they are presented within an experiential learning model.
